# Fracture Resistance of a Bone-Level Two-Piece Zirconia Oral Implant System—The Influence of Artificial Loading and Hydrothermal Aging

**DOI:** 10.3390/jfb15050122

**Published:** 2024-05-07

**Authors:** Ralf J. Kohal, Ellen Riesterer, Kirstin Vach, Sebastian B. M. Patzelt, Aljaž Iveković, Lara Einfalt, Andraž Kocjan, Anna-Lena Hillebrecht

**Affiliations:** 1 Medical Center—University of Freiburg, Center for Dental Medicine, Department of Prosthetic Dentistry, Faculty of Medicine, University of Freiburg, 79106 Freiburg, Germany; ellen.riesterer@uniklinik-freiburg.de (E.R.);; 2 Medical Center—University of Freiburg, Institute of Medical Biometry and Statistics, Faculty of Medicine, University of Freiburg, 79104 Freiburg, Germany; kirstin.vach@uniklinik-freiburg.de; 3 Private Dental Clinic, 78658 Zimmern ob Rottweil, Germany; 4 Department for Nanostructured Materials, Jožef Stefan Institute, Jamova 39, 1000 Ljubljana, Slovenia; aljaz.ivekovic@ijs.si (A.I.); lara.einfalt@ijs.si (L.E.);; 5 Jožef Stefan International Postgraduate School, Jamova 39, 1000 Ljubljana, Slovenia

**Keywords:** artificial chewing simulation, dental implants, loading/aging, zirconia, two-piece, stability, abutment screw

## Abstract

Preclinical and clinical research on two-piece zirconia implants are warranted. Therefore, we evaluated the in vitro fracture resistance of such a zirconia oral implant system. The present study comprised 32 two-piece zirconia implants and abutments attached to the implants using a titanium (*n* = 16) or a zirconia abutment screw (*n* = 16). Both groups were subdivided (*n* = 8): group T-0 comprised implants with a titanium abutment screw and no artificial loading; group T-HL was the titanium screw group exposed to hydro-thermomechanical loading in a chewing simulator; group Z-0 was the zirconia abutment screw group with no artificial loading; and group Z-HL comprised the zirconia screw group with hydro-thermomechanical loading. Groups T-HL and Z-HL were loaded with 98 N and aged in 85 °C hot water for 10^7^ chewing cycles. All samples were loaded to fracture. Kruskal–Wallis tests were executed to assess the loading/bending moment group differences. The significance level was established at a probability of 0.05. During the artificial loading, there was a single occurrence of an implant fracture. The mean fracture resistances measured in a universal testing machine were 749 N for group T-0, 828 N for group Z-0, 652 N for group T-HL, and 826 N for group Z-HL. The corresponding bending moments were as follows: group T-0, 411 Ncm; group Z-0, 452 Ncm; group T-HL, 356 Ncm; and group Z-HL, 456 Ncm. There were no statistically significant differences found between the experimental groups. Therefore, the conclusion was that loading and aging did not diminish the fracture resistance of the evaluated implant system.

## 1. Introduction

Zirconia oral implants represent an additional aspect in modern implant dentistry, offering an addendum to traditional tooth replacement solutions which use titanium implants [1,2,3,4]. By harnessing the unique properties of zirconia, these implants could provide patients with aesthetically pleasing, long-lasting, and biologically compatible implant restoration options. For centuries, addressing the issue of missing teeth has posed a fundamental challenge in dentistry. Traditional oral implants predominantly utilize titanium as the implant material, a choice that has demonstrated success in restoring both oral function and aesthetics [5,6,7,8]. Titanium oral implants have been widely used and are generally considered safe and effective for tooth replacement. However, like any medical procedure or material, there are some disadvantages and potential drawbacks associated with titanium implants. While rare, some individuals may develop allergic reactions to titanium implants [9,10,11,12]. This can lead to localized inflammation, discomfort, and even implant failure. Some individuals may be sensitive to metals in general, occasionally extending to titanium. This sensitivity can also lead to discomfort and complications. Hypersensitivity to titanium is uncommon but is a known drawback [13,14]. Titanium implants are generally corrosion-resistant; however, in certain conditions, corrosion can occur [15,16,17,18,19,20]. Exposure to possibly corrosive substances, such as toothpastes [21,22], acidic mouthwashes, or other cleaning methods [23], as well as to an increased bacterial load, [19,24,25,26] may lead to titanium corrosion and in turn may influence the biofilm composition and tissue damage.

Finally, titanium implants, due to their metallic nature and gray color, may not be as aesthetically pleasing as tooth-colored materials like zirconia. This is particularly important for implants placed in the aesthetic area with thin soft and hard tissues and where aesthetics is a major concern. 

It is important to note that many of these disadvantages are relatively rare or can be mitigated with proper patient selection, careful treatment planning, and regular follow-up care. Patients considering oral implants should discuss these potential disadvantages with their dentist or oral surgeon to make informed decisions about their treatment options. Additionally, advancements in oral implant materials and techniques may address some of these drawbacks in the future.

Nevertheless, the quest for materials offering potentially enhanced biocompatibility and aesthetics has spurred the emergence of zirconia oral implants. These implants are produced from zirconium dioxide (ZrO_2_), a ceramic material characterized by its crystalline structure. The composition for biomedical applications can vary, for example, yttria-stabilized (~3 mol. %) tetragonal zirconia polycrystal (TZP) or alumina-toughened zirconia (ATZ), impacting the implant’s properties and performance. Zirconia’s good biocompatibility arises from its chemical inertness, preventing adverse reactions with the surrounding soft and hard tissues [27,28,29]. Investigations have revealed that zirconia oral implants elicit minimal inflammatory responses and are well-tolerated by the human body [30,31,32]. Successful osseointegration is important for the long-term success of oral implants. Modern zirconia implants promote osseointegration through their biocompatibility and tailored surface characteristics, which support bone cell adhesion and proliferation on their surfaces [32,33]. The aesthetic appeal of zirconia oral implants is a notable advantage over traditional titanium implants. Zirconia’s natural tooth color and translucency closely resemble those of natural teeth, resulting in a positive integration with the patient’s smile. Patient satisfaction with zirconia oral implants related to aesthetics and function is moderate to high [34]. Hence, zirconia implants might be preferred, especially in the aesthetic region, if they demonstrate comparable rates of survival and success to titanium implants. Clinical studies and systematic reviews have shown promising outcomes for zirconia oral implants [1,2,3,4]. Research indicates comparable success rates between one-piece zirconia and titanium implants in single-tooth replacements and three-unit reconstructions [35]. In the case of one-piece implants, the mechanical characteristics of zirconia, including its elevated flexural strength and resilience to fracture, enhance its appropriateness as a material for oral implants. In single-piece systems, post-implantation axial correction of the abutment is only marginally achievable through abutment preparation and is associated with diminished stability. Furthermore, single-piece implants are directly exposed to masticatory forces, transmitting them to the bone without reduction [36]. In the case of two-piece systems, axial corrections can be implemented during prosthetic treatment, and the prosthetic structure can be replaced or repaired based on specific indications, but scientific data are still weak in the clinical as well as preclinical area [37,38]. An undesirable complication is the fracture of the connecting screw. Screws made of titanium, gold, or carbon fiber-reinforced polymers are available for this purpose. A significant advantage in the context of a completely metal-free restoration in two-part systems would be if the screw between the implant and abutment could be manufactured from the same material as the implant and abutment. The two-piece implant system Z-Systems introduces, for the first time, a ceramic abutment screw thereby presenting a truly all-ceramic implant system. The question arises as to whether the offered system, with the ceramic abutment screw, can withstand long-term loading and, consequently, serve as an alternative to the titanium abutment screw.

Hence, the objective of the current study was to preclinically assess the fracture resistance of an innovative two-piece bone-level zirconia oral implant system (Z5-BL, Z-Systems) with two different abutment screws (titanium versus zirconia screw) under artificial functional load. The innovation and significance of the study lie in the exploration and development of a two-piece zirconia oral implant as a potential alternative to traditional two-piece titanium implants in implant dentistry. The study specifically focuses on a two-piece bone-level zirconia oral implant system, introducing a novel element—the use of a ceramic abutment screw—marking a transition towards a completely metal-free, all-ceramic implant system. The investigation’s significance lies in its aim to evaluate preclinically the fracture resistance of this innovative zirconia oral implant system, comparing two different abutment screws (titanium versus zirconia) under artificial functional load. The outcomes of this investigation have the potential to contribute valuable insights into the feasibility and durability of zirconia implants.

## 2. Materials and Methods

### 2.1. Experimental Setup and Investigated Implants

A total of thirty-two two-piece zirconia implants were incorporated in the study. For 16 implants, the zirconia abutments (BL-CB2545, Z-Systems) were connected with a titanium screw (BL-OST-H, Z-Systems) (group T, for titanium screw), and for 16 implants, the abutments were fixed with a zirconia screw (BL-OSC-H, Z-Systems) (group Z, for zirconia screw). Each group was subdivided into subgroups of eight implants depending on whether the implants were submitted to hydrothermal aging and mechanical loading (HL) in an artificial chewing simulator or not (0). Thus, four subgroups resulted: T-0, T-HL, Z-0, and Z-HL. One sample from each group was chosen at random, cross-sectioned, and examined for its crystal composition using scanning electron microscopy (SEM). Apart from these samples, all other specimens that endured the dynamic loading and aging were subjected to a quasi-static loading test using a universal testing machine until fracture occurred. The findings were then subjected to statistical analysis.

### 2.2. Experimental Zirconia Implant

**Figure 1:**
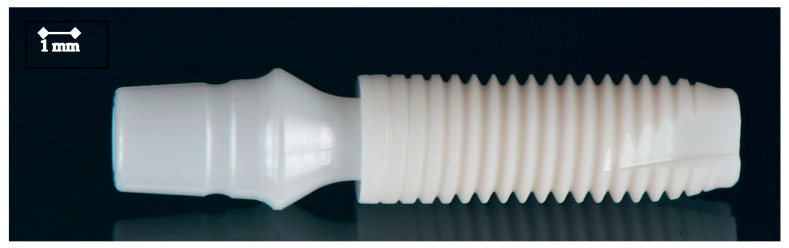
The investigated zirconia oral implant with the attached zirconia abutment.

For this in vitro investigation, 32 two-piece bone-level zirconia oral implants (Z5-BL-4012, Z-Systems, Oensingen, Switzerland) were used (Figure 1). The implants were fabricated from Y-TZP-A powder (with ≥99.0 m% ZrO_2_ + HfO_2_ + YO_3_; >4.5–≤6.0 m% Y_2_O_3_; ≤5 m% HfO_2_; ≤0.5 m% Al_2_O_3_ and ≤0.5 m% other oxides) with a mean grain size of 0.4 µm. The implants measured 4 mm in diameter and 12 mm in length. The implants featured a screw-shaped design with tapered threads and a widened core diameter at the upper portion of the thread. According to the manufacturer, they were produced using cold isostatic pressing, hot isostatic compaction, sintering, and finally, a hot isostatic postcompaction process (HIP). Then, following the HIP process, the material underwent a hard machining process, and finally, the implant surface was air-abraded using corundum and subjected to laser modification (SLM^®^; surface laser modified; Z-Systems), resulting in a surface roughness (Ra) ranging from 0.4 to 0.55 µm. The details of the process, however, are a company secret. The implants possessed a v-shaped thread pattern with rounded edges, distinguished by symmetrical parallel grooves at the tops of the threads. The implants were plasma-sterilized. In addition, the zirconia abutments were similarly produced (except surface treatment) and had a gingival height of 2.5 mm and a diameter of 4.5 mm.

**Figure 2:**
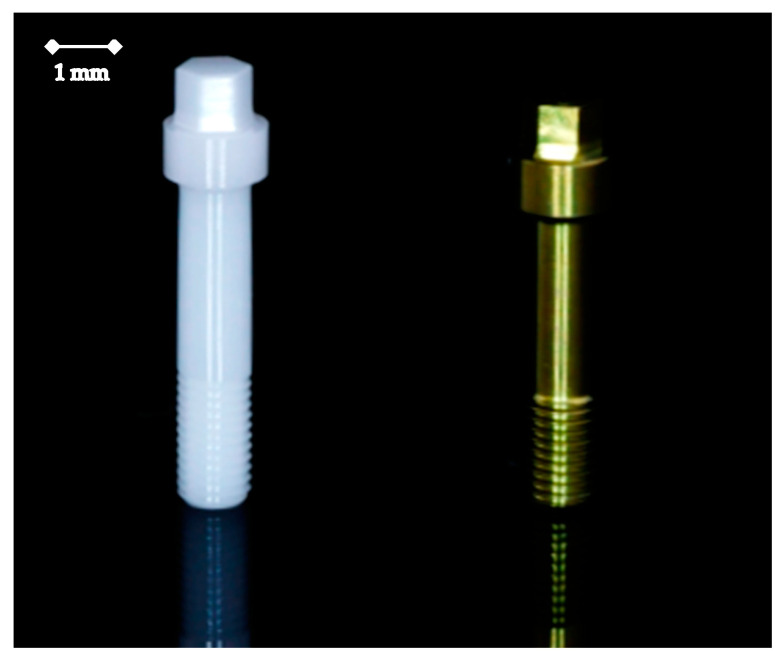
Left: Zirconia abutment screw (BL-OSC-H). Right: Titanium abutment screw (BL-OST-H).

The zirconia abutments (BL-CB2545, Z-Systems) were screw-retained in the implant with either a titanium (BL-OST-H, Z-Systems) or a ceramic abutment screw (BL-OSC-H, Z-Systems) (Figure 2). According to the manufacturer’s specification, the ceramic abutment screw had a diameter of 1.2 mm.

**Figure 3:**
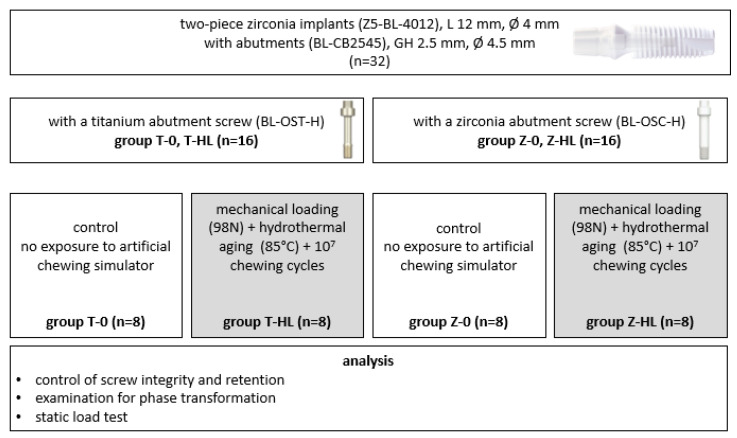
Experimental setup of the investigation. L = length of the implants; GH = gingival height of the abutment; T = titanium; Z = zirconia; 0 = no exposure to the chewing simulator; HL = hydrothermal aging and mechanical loading in the chewing simulator.

A scheme of the experimental setup is presented in Figure 3.

### 2.3. Preparation of Samples for Artificial Loading and Hydrothermal Aging in the Artificial Chewing Simulator

To simulate the functional loading of the two-piece implant system under artificial aging conditions, the experimental setup was selected according to ISO standard 14801 [39] and implemented as follows: all implants were embedded in sample holders made of Polyether-Ether-Ketone (PEEK), which had a height of 20 mm, an outer diameter of 16 mm, and an inner diameter of 7 mm. The interior of each embedding tube was equipped with three small recesses, serving as rotational protection. Additionally, on the outer surface of the tube, the sample name was engraved as a recognition feature, along with a notch. This notch corresponded to the direction from which the loading forces acted on the samples (tension side). Furthermore, the inner side of the PEEK tubes was roughened with a sandblaster using Al_2_O_3_ (0.5 bar, particle size of 50 µm). The height of the implants in the PEEK tubes could be varied with a set screw in the base of the tube. The height of the implant-abutment connection plus the loading hemisphere was adapted with this screw according to ISO standard 14801 in such a way that the loading center of the implants was 11 (±0.5) mm above the upper edge of the embedding sleeve, including a 3 mm simulated bone recession. The loading angle to the vertical implant axis was 30 (±2) degrees, which led to a 5.5 (±0.5) mm lever arm. The implants were fixated in the PEEK with a dual-polymerizing acrylic material (LuxaCore^®^ Dual-Automix, DMG, Hamburg, Germany). 

**Figure 4:**
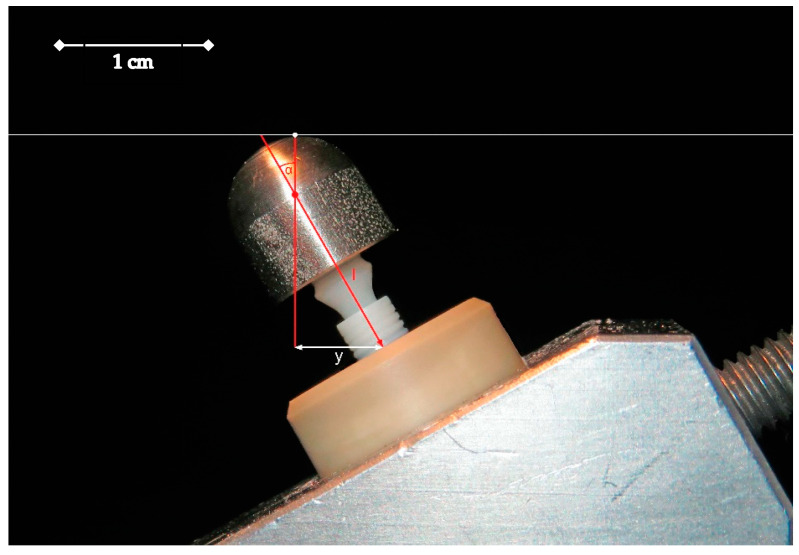
A sample (implant with screw-retained abutment plus loading hemisphere) embedded according to ISO standard 14801 in a PEEK tube (y = lever arm, 5.5 mm; I = distance from tube rim to the loading center, 11 mm; α = embedding angle, 30°).

The dual-polymerizing acrylic exhibits an elastic modulus of 8 GPa, approximately corresponding to the elastic modulus of human bone [40]. After fixating the implants in the tubes, the zirconia abutments were inserted and the abutment screws were attached. Subsequently, the screws were tightened using a ratchet to a final torque of 15 Ncm. Each embedded specimen was subsequently photographed in a standardized manner and the respective distances were measured using an image editing program (Adobe Photoshop CS 6, Adobe, San Jose, CA, USA) (Figure 4).

### 2.4. Mechanical Loading and Hydrothermal Aging

The T-HL and Z-HL specimens were affixed onto a computer-regulated chewing simulator (CS-4.8, SD-Mechatronik, Feldkirchen-Westerham, Germany) using tube holders fabricated from aluminum. Dynamic loading, exerting a force of 98 N on the samples, resulting in a bending moment of approximately 54 Ncm, was applied using a stainless-steel antagonist featuring a flat surface for 10 million cycles (at a frequency of 1.3 Hz). For hydrothermal aging, the chambers containing the specimens were filled with distilled water maintained at a constant temperature of 85 °C via an integrated heating system. To prevent water evaporation, an automatic refill system was employed. Each chewing cycle comprised a vertical load (60 mm/s) followed by a horizontal movement of 0.5 mm (55 mm/s), replicating physiological mastication [41]. Throughout the testing period, the specimens were inspected twice daily for any signs of fractures or abutment loosening. The implants of groups T-0 and Z-0 (control groups) were neither exposed to loading nor to aging. 

### 2.5. Phase and Microstructural Composition Beneath the Surface

Prior to analyses, the complete implant specimens (without the loading hemisphere) were embedded in epoxy resin (EpoFix, Struers, Ballerup, Denmark) and subsequently cut in half using a sectioning saw equipped with a 0.4 mm diamond blade (IsoMet Blade 15LC, Buehler, Leinfelden-Echterdingen, Germany) in a cutting solution (IsoCut Fluid, Buehler, Leinfelden-Echterdingen, Germany). The blade speed utilized was 150 rpm and the load applied to the blade was 100 g. After cutting in half, the cut surface was ground with diamond plates and silicon carbide papers. The final step was polishing with a 1 μm fine diamond paste (Struers Tegramin-30, Struers). 

**Figure 5:**
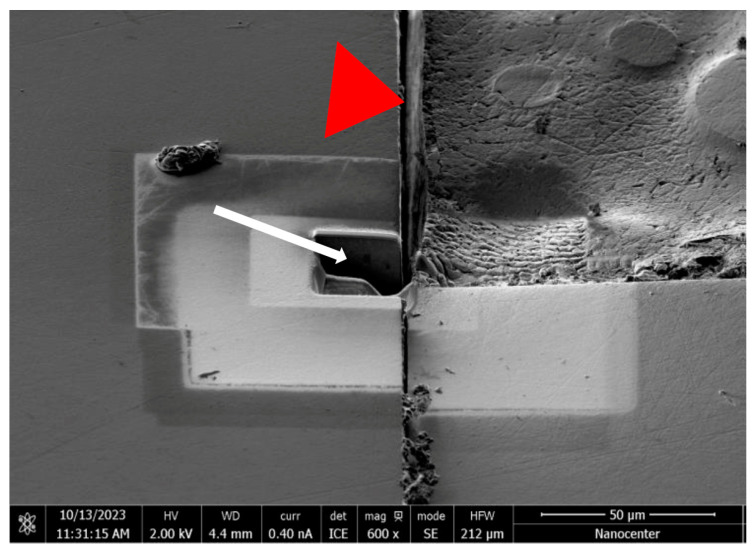
An ion-milled cross-section (white arrow) prepared by FIB in the implant head (red arrow head) of a loaded hydrothermally aged sample.

The subsurface microstructural features of the implants were analyzed on ion-milled cross-sections prepared with a focused ion beam scanning electron microscope (Helios NanoLab 650, FEI, Hillsboro, OR, USA; Figure 5). 

The region of interest for the thermomechanically treated groups was situated on the tensile side. To prevent any curtaining effect during milling, a 2.5 µm thick tungsten coating was applied to the surface of interest by means of an ion-beam-assisted gas-injection system at 30 kV and 0.77 nA. Focused ion beam (FIB) milling was conducted across the designated interface regions using an ion beam at 30 kV and 65 nA, followed by ion polishing with 21 nA and 9.5 nA. The ion-milled areas were analyzed at an angle of 52° with an electron probe at 2 kV and 0.4 nA. For Raman spectroscopy, an NTEGRA Spectra NT-MTD (SPECTRUM INSTRUMENTS LTD., Limerick, Ireland) was used to analyze the distribution of monoclinic and tetragonal phases in the zirconia implant specimens. A laser with a beam of 633 nm was applied as the excitation source, using a power of 3 mW. Four line analyses were performed per specimen at four different positions (two in the abutment and two in the implant areas) of each sample to determine the presence of the monoclinic phase. Starting from the edge of the polished surface sample, with line distances of 0, 1, 2, 3, 4, 5, and 10 μm going inward towards the implant center, the displacement of the measurements was 1 μm vertically in depth. The diameter of the interaction cross-section was estimated from the numerical aperture (NA; 0.7) of the objective and was approximately the excitation wavelength. The time of each acquisition was 50 s. The spectra captured were then normalized and the peak ratio was calculated from the intensity of the characteristic peaks for the monoclinic (178 cm^−1^) and tetragonal (260 cm^−1^) zirconia. The arithmetic mean value and standard deviation at each distance were calculated from the four different line measurements per sample.

The grain size of the implant material was assessed with the linear-intercept method, not using a correction factor. The intercept dimension of the grains was determined from at least three FIB-SEM micrographs.

### 2.6. Quasi-Static Loading Assessment

To evaluate fracture resistance, seven out of eight specimens from each group were placed in a universal testing machine (ZwickRoell Z010, ZwickRoell AG, Ulm, Germany) at a 30° angle to the loading axis and subjected to loading until fracture occurred. The crosshead speed of the machine was maintained at 10 mm/min. The endpoint of the assessment was defined by the sudden decline in load capacity.

### 2.7. Statistical Analyses

For statistical analyses, due to the non-normal distribution of the data, a Kruskal–Wallis test was used for group comparisons of fracture resistance as well as bending moments. Dunn tests with Holm correction for multiple comparisons were used for subsequent pairwise comparisons. The level of significance was defined at 5% (STATA 17.0, StataCorp LP, College Station, TX, USA).

## 3. Results

### 3.1. Mechanical Loading and Hydrothermal Aging Assessment

The specimens underwent dynamic loading with a force (F) of 98 N (equivalent to 10 kg), resulting in a bending moment of 54 Ncm. With the exception of one specimen from the T-HL group, all survived 10 million cycles of loading and aging at 85 °C without experiencing fractures or abutment screw loosening. The fracture load and bending moment of the sample that fractured in the chewing simulator was taken into statistical account with 98 N (54 Ncm).

### 3.2. Phase and Microstructural Composition Beneath the Surface

The implants exhibited a linear intercept grain size value of 390 ± 40 nm.

**Figure 6:**
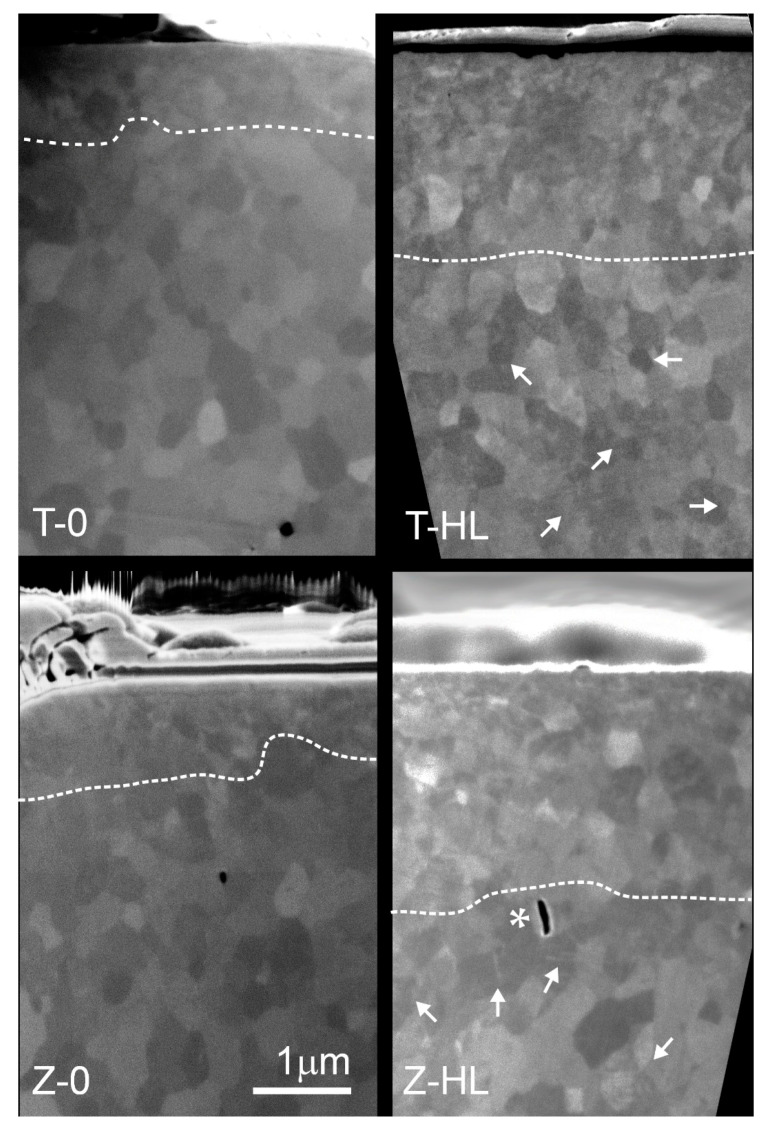
SEM micrographs of cross-sections showing a subsurface region of ∼5 µm in depth. White dotted lines roughly indicate the transformed region. The white arrows indicate distinct altered individual grains containing intragranular domains and monoclinic variants with lath-like geometry. Transformation changes gradually become less pronounced going deeper into the specimen. The non-loaded/non-aged groups (T-0, K-0) showed a 1 µm deep and the loaded and hydrothermally aged groups (T-HL, K-HL) an approximately 3 µm deep transformation. * in Z-HL: During microstructural inspection with FIB-SEM several distinct and isolated pores were detected (as expected), representing the residual porosity of the dense zirconia implant material. The pores had a low coordination number (≤5) and were in the size range of a single grain (~300 nm). As such, these pores usually do not act as critical flaws initiating failure. Pore sizes and/or surface and volume flaws in the size range 5–10 µm or larger are critical flaws initiating a crack/failure under stress.

The subsurface regions of the implants without and with mechanical and hydrothermal loading were evaluated by FIB-SEM analysis (Figure 6). Without combined treatment, the implants revealed an approximately 1 μm thick transformation zone depth affecting ~2 grain columns with a refined, nanoscale intergranular domain microstructure. With mechanical load and hydrothermal aging, the transformed zone depth became larger, extending approximately 3 μm into the bulk. The transformed layer was coherent, i.e., exhibiting indistinct boundaries between affected and unaffected regions, without the presence of microcracks typical for low-temperature degradation (LTD). At even deeper regions, beyond 3 μm, distinct intragranular boundaries and twin-related monoclinic variants with lath-like geometry could be observed as a result of thermomechanical-induced transformation.

**Figure 7:**
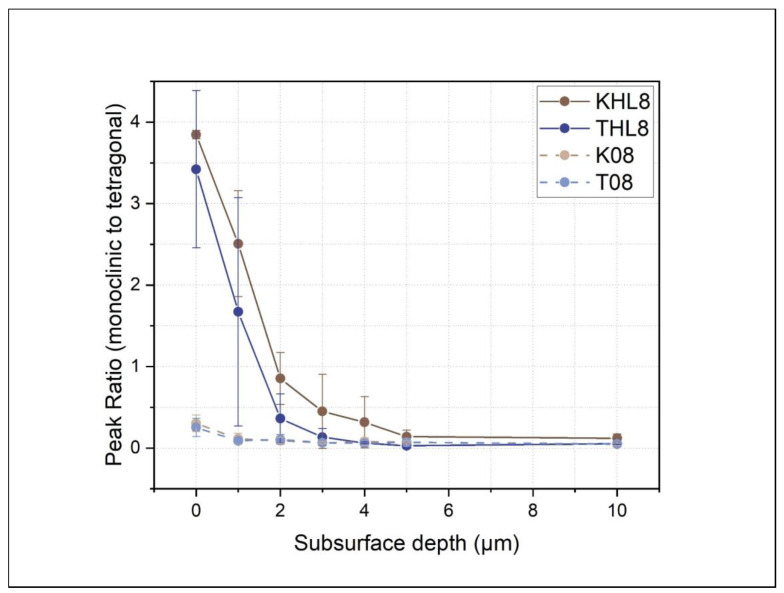
Raman depth profiles of the monoclinic-to-tetragonal phase ratios between the different groups based on the peak intensities at 178 cm^−1^ (monoclinic) and 260 cm^−1^ (tetragonal) for the AB samples before and after thermomechanical loading.

Raman spectroscopy was employed to evaluate the phase transformation from tetragonal to monoclinic of the implant specimens as a result of thermomechanical loading. In Figure 7, the line depth profiles showing arithmetic mean values with standard deviations of the monoclinic/tetragonal phase proportion are presented. Prior to thermomechanical treatment, only a minimal quantity of the monoclinic phase was detected, located on the implant surface, up to 1 μm in depth. Thermomechanical loading induced a subsurface phase transformation from tetragonal to monoclinic (t-m) that was most significant in the topmost 2 μm zone depth. Afterwards, the presence of a monoclinic phase is gradually lowered to the detection limit at 5 μm depth. The extent of transformation in specimens with and without thermomechanical loading observed by Raman spectroscopy is well in line with the FIB-SEM observations.

### 3.3. Quasi-Static Loading Assessment

The average load required to cause fracture was 749 N (SD: 208 N) for the control group T-0, 652 N (SD: 256 N) for the T-HL group, 828 N (SD: 226 N) for group Z-0, and 826 N (SD: 114 N) for the Z-HL group (Kruskal–Wallis test: *p* = 0.477). The average bending moments leading to fracture were 411 Ncm (SD: 120 Ncm) for group T-0, 356 Ncm (SD: 139 Ncm) for group T-HL, 452 Ncm (SD: 124 Ncm) for group Z-0, and 456 Ncm (SD: 62 Ncm) for group Z-HL (Kruskal–Wallis test: *p* = 0.416) (Table 1).

## 4. Discussion

It was proposed that new materials for reconstructive dentistry should be evaluated in vitro first regarding their stability and performance [42]. Since a two-piece implant system with a ceramic screw is not yet available, there is no data on the resistance and load-bearing capabilities of such a full-zirconia system. The tested zirconia oral implant system is the first, and currently the only, available implant system with a zirconia abutment screw. 

Recently, our group presented the results of another two-piece zirconia implant system [41]. Although, also being a two-piece system, the system from the former investigation [41] was per se not a bone-level system and the abutment screw was made from carbon-reinforced PEEK (Zeramex XT, Dentalpoint AG, Spreitenbach, Switzerland). The system of the present investigation was a two-piece bone-level system evaluated either with a titanium or a zirconia abutment screw (Z-Systems). In addition, the implants of the former investigation were fabricated from alumina-toughened zirconia (ATZ) and showed a tapered macro-design, the implants from the present study were made from 3Y-TZP-A and had a parallel cylindrical design. Furthermore, the abutment–implant connection was a butt-joint connection in the former investigation and a platform-switching connection in the present study, However, the in vitro experimental setups in both investigations were similar in order to be able to compare the outcomes between the different implant systems [41].

We observed—in the present investigation—fracture resistance values for the zirconia abutment screws that were comparable to the titanium abutment screws in the present investigation. Furthermore, the in vitro simulation of aging processes (to which the implant system would be exposed in the oral cavity) revealed no significant material damage, either when using the zirconia screw or the titanium screw.

The findings from the present quasi-static loading tests, with or without chewing simulation, in this study revealed bending moments resulting in catastrophic failure for the various groups ranging from 356 to 456 Ncm. The maximum values were observed in the control groups without thermomechanical treatment, whereas the group subjected to aging and loading exhibited lower values. Nonetheless, these values were significantly higher than the bending moment range of 5–95 Ncm obtained clinically with strain gauges in implant-retained bridges [43]. The principal results of the current study revealed no discernible impact of (hydrothermal) aging and loading on the fracture resistance of the examined implant system. Moreover, our study findings suggest that the stability of both abutment screws is sufficiently robust to endure prolonged exposure to normal occlusal forces [38]. The fracture resistance of the zirconia implant appeared capable of withstanding the normal bite forces exhibited by patients, with the highest measured in vivo bending moment during biting reaching 95 Ncm [43]. When adding the same amount of bending moment, a minimum fracture resistance of approximately 200 Ncm is identified and regarded as adequate for clinical safety [38]. This fracture resistance was consistently observed in all groups tested in our study.

All specimens within the Z-HL and T-HL groups successfully withstood the dynamic loading test and presented no observable failings at the implant/abutment interfaces and no loosening of the abutment/implant connections occurred. The utilization of 10 million chewing cycles aligns with frequently cited parameters in the literature for assessing the fracture resistance of zirconia oral implants [44,45,46,47]. Given the heterogeneous outcomes in studies evaluating annual chewing contacts, the 10 million chewing cycles employed in the present investigation paralleled a clinical timeframe spanning from 12.5 up to 40 years [48,49].

To ensure a reliable strength of two-piece implant systems, the configuration of the connection between the implant and abutment plays a crucial role. Particularly, the stability of the connecting screw is a vital factor for the integrity of the implant system under the expected clinical loads [37]. Titanium screws showed reliable results, but do not enable a completely metal-free workflow like one-piece implant systems. To enable a completely metal-free workflow in two-piece implant systems, there are some systems using carbon fiber-reinforced PEEK screws. Strictly speaking, no available two-piece zirconia implant systems qualify as “all-ceramic, full-zirconia” implant systems due to the utilized screw material. The implant system investigated in this study is the first system available on the market with a zirconia abutment screw.

For comparability with former in vitro studies assessing zirconia implants, the implants in this current investigation, akin to others, were fixed according to the ISO 14801 standard. In an effort to replicate the clinical scenario, a resin material with a Young’s modulus of about 3 GPa, resembling the modulus of natural bone, was employed [40]. Additionally, a bone recession of 3 mm was replicated, although it should be noted that this value exceeds the peri-implant bone loss reported in clinical studies [1,4].

Artificial aging, in a humid environment at elevated temperatures, has the potential to induce spontaneous t-m transformations in Y-TZP. This phenomenon is known as LTD [50,51]. Despite the fact that the precise mechanism of LTD has not been fully elucidated, it appears that water molecules become incorporated into the polycrystalline structure, triggering the start of the transformation from tetragonal to monoclinic variants within the affected grain. The resultant water derivatives instigate the transformation from tetragonal to monoclinic, leading to a volumetric expansion of 4% [52]. Each enlarging grain stresses the adjacent material, causing microcracking and facilitating further water penetration [53]. The so-called stress corrosion process that initiates at the surface and advances into the bulk may contribute to a decline in the mechanical performance of zirconia within the oral cavity [54]. Notably, zirconia implants produced from Y-TZP are susceptible to LTD that occurs in vivo as well [55]. However, the material’s linear aging kinetics depend on several microstructural aspects such as grain size, phase partitioning, and surface finish [56]. On the other hand, zirconia–alumina composites (alumina-toughened zirconia) are not as prone to this degradation process [53].

The as-received two-piece zirconia implant system evaluated in the present study already exhibited a thin transformed layer prior to the thermomechanical loading (Figure 7). The 1 μm thick layer consisted of refined, nanoscaled (transformed) grains (Figure 6), the origin of which could be related to the air-particle abrasion and surface laser modification provided by the manufacturer. Similar features in the topmost layers were previously observed in mechanically invasive surface treatments such as grinding, air-particle abrasion, and/or impacting [56,57,58,59]. The latter could trigger surface alterations and the development of compressive micro-residual strains [60].

After thermomechanical loading with 10 million chewing cycles, the transformed subsurface layer roughly doubled in thickness. Considering the approximated clinical timeframe, spanning from 12.5 up to 40 years, simulated by 10 million cycles, the 1–2 μm gain in the transformed surface layer does not seem critical. The overall 2.5–3 µm thick transformed (t-m) layer would correspond to an approximately 40% XRD monoclinic fraction (Xm), which, according to in vitro–in vivo extrapolations, would reflect potentially ≥30 years of clinical service [61]. The in vivo studies, however, imply even faster aging kinetics [55]. The absence of microcracks in the transformed layer, which are usually present in the aged, transformed subsurface layer, could well point to the presence of compressive micro-residual stresses, which were shown to slow down the aging kinetics and increase the fracture strength [56,59,62]. The transformation can be (partly) reverted and stresses annihilated by low-temperature annealing through regeneration firing or, possibly, by laser treatment [59,63].

Y-TZP materials show high chemical and electrical resistance, opening up promising application scenarios for roller bearings in corrosive environments or in systems subjected to electrical current flow, such as in wind turbine generators. Additionally, zirconia ceramics are furthermore applied as thermal barrier coatings in modern gas turbines and jet engines. The flexural strength (1200 MPa), the fracture toughness (8 MPa√m), and the elastic modulus (210 GPa) of the present implant material are in the range of the values found for zirconia materials utilized in extreme conditions like thermal barrier coatings (elastic modulus: 95–142 GPa; fracture toughness: 1.46–5.49 MPa√m) [64] pointing towards a stable, long-lasting material for intraoral biomedical application. Zirconia implants, based on yttria-stabilized tetragonal zirconia, are medical devices that need to comply with ISO 13356:2015 [65] if sold on the market. The standard carefully specifies the requirements (minimum strength for example) and respective test means for a ceramic bone substitute for utilization as a substrate for implants.

The current study faces limitations inherent in its in vitro design, as it can only provide an approximation of clinical conditions. Such simulations, while useful, cannot fully replicate the complexities of real-world scenarios, including precise chewing patterns, temperature fluctuations, biomechanical variations, and other clinical factors. Additionally, the study’s sample size is relatively small, a common issue in similar research endeavors [38]. Nonetheless, despite these limitations of such an investigational setup, the findings from these tests suggest that zirconia implants may withstand physiological loads, as indicated by previous in vitro investigations [38]. Enhancing the fidelity of artificial chewing simulations to better mimic real-world conditions represents a promising avenue for future research in this field.

## 5. Conclusions

Based on the constraints of the current study, it can be inferred that artificial loading and hydrothermal aging do not diminish the fracture resistance of the examined implant system. Both the zirconia screw and the titanium screw proved to be sufficiently resilient. The fracture stability and bending moments of the evaluated implants are similar to those of other two-piece implants, whether made of zirconia or titanium. From a stability perspective, this implant system demonstrates the capacity to endure prolonged clinical loading and the results of our investigation suggest a stable system that possibly includes a favorable clinical performance. However, clinical investigations assessing the comprehensive performance of two-piece zirconia implants are necessary to detect any possible technical or biological deficiencies.

## Figures and Tables

**Table 1:** Mean values ± standard deviations (median) of the implant fracture loads (N) and bending moments (Ncm) of the experimental groups.

Group	Number of Specimens	Fracture Resistance [N] (Median)	Bending Moment [Ncm] (Median)
T-0	7	749 ± 208 ^a^ (767)	411 ± 120 ^a^ (422)
T-HL	7	652 ± 256 ^a^ (701)	356 ± 139 ^a^ (387)
Z-0	7	828 ± 226 ^a^ (736)	452 ± 124 ^a^ (408)
Z-HL	7	826 ± 114 ^a^ (860)	456 ± 62 ^a^ (474)

Groups with identical superscript letters were statistically not significantly different with respect to pairwise comparisons within a column.

## Data Availability

The data presented in this study are available on request from the corresponding author (accurately indicate status).
